# Lung organoids for respiratory diseases: overcoming translational hurdles in drug discovery and safety assessment

**DOI:** 10.3389/fbioe.2026.1859058

**Published:** 2026-06-15

**Authors:** Xiaoya Mao, Minmin Cai, Bimeng Fan

**Affiliations:** 1 Department of Pharmacy, The Affiliated People’s Hospital of Ningbo University, Ningbo, China; 2 Department of Anesthesiology, The Affiliated People’s Hospital of Ningbo University, Ningbo, China

**Keywords:** bioengineering, drug discovery, lung organoids, predictive toxicology, respiratory diseases

## Abstract

Respiratory diseases, encompassing chronic inflammatory conditions, interstitial fibrotic disorders, acute infectious diseases, and pulmonary malignancies, represent a profound global health burden with unacceptably high morbidity and mortality rates. Historically, the pharmaceutical pipeline for respiratory therapeutics has suffered staggering attrition rates during clinical development. This is primarily due to the fundamental inability of conventional two-dimensional cell cultures and *in vivo* animal models to faithfully recapitulate the complex three-dimensional architecture, multicellular heterogeneity, and human-specific physiological dynamics of the pulmonary system. To bridge this critical translational gap, lung organoids—self-organizing, three-dimensional microphysiological constructs derived from pluripotent or adult stem cells—have emerged as a useful human-cell-based platform. This comprehensive review critically evaluates current lung organoid technologies, elucidating their derivation pathways and capacity for high-fidelity disease modeling. We analyze their application in dissecting the pathogenesis of chronic obstructive pulmonary disease, idiopathic pulmonary fibrosis, viral infections including SARS-CoV-2, and non-small cell lung cancer. Furthermore, we highlight their important role in predictive toxicology for assessing environmental inhalation hazards, cosmetic safety, and drug-induced lung injury, aligning with evolving regulations prioritizing alternatives to animal testing. Despite their immense potential, widespread clinical and industrial translation is currently impeded by biological bottlenecks: the absence of functional vascularization, incomplete immune integration, and reliance on undefined xenogeneic matrices. We systematically examine bioengineering strategies addressing these limitations—including synthetic hydrogels, microfluidic organ-on-a-chip platforms, and 3D bioprinting—to overcome translational hurdles, accelerate precision medicine, and improve respiratory pharmacology.

## 1 Introduction

The respiratory system represents a major physiological interface between the internal physiological environment and inhaled external factors, rendering it uniquely susceptible to a multitude of severe pathologies (Bosáková et al., 2022; Kaimakamis and Chasapidou, 2022). Respiratory disorders, ranging from chronic inflammatory syndromes such as asthma and chronic obstructive pulmonary disease (COPD) to aggressive interstitial lung diseases like idiopathic pulmonary fibrosis (IPF), afflict hundreds of millions of individuals worldwide (Bosáková et al., 2022; Incalzi and Fimognari, 2023). This immense clinical burden is further compounded by the continuous emergence of highly transmissible airborne pathogens, vividly exemplified by the severe acute respiratory syndrome coronavirus 2 (SARS-CoV-2) pandemic, as well as rising global levels of anthropogenic environmental pollutants and an aging demographic (Li et al., 2021; Bosáková et al., 2022; Guiot et al., 2023). Despite intensive, decades-long research efforts and immense capital investment, our comprehensive understanding of lung biology and its dynamic interaction with disease processes remains critically incomplete, resulting in a profound translational gap in the development of efficacious therapeutic interventions (Bosáková et al., 2022; Chu et al., 2026).

Historically, preclinical biomedical research has relied almost exclusively on two-dimensional (2D) immortalized cell lines and *in vivo* mammalian models (Shrestha et al., 2023; Yang et al., 2023; Zimmerling et al., 2024; Li et al., 2025a). While 2D monolayer cultures provide highly scalable, standardized, and cost-effective platforms for high-throughput initial compound screening, they fundamentally lack the complex cellular heterogeneity, precise spatial organization, cell-to-cell communication networks, and dynamic biomechanical microenvironments characteristic of native human lung tissues. Conversely, animal models, though possessing systemic physiological complexity, frequently fail to accurately predict human pharmacological responses and toxicological susceptibilities due to deeply entrenched evolutionary divergences in genetics, pulmonary anatomy, and immunological repertoires (Chen and Na, 2022; Zhang et al., 2025a). These biological discrepancies contribute to high attrition during clinical development, although failure rates vary substantially by therapeutic area, disease indication, and clinical phase (Singh et al., 2025).

In direct response to these critical methodological and physiological limitations, three-dimensional (3D) organoids have emerged as a revolutionary and disruptive paradigm in cellular biology and translational medicine (Farhat et al., 2021; Zhang et al., 2025a). Lung organoids are defined as self-organizing, self-renewing, and spatially structured cellular constructs derived either from human pluripotent stem cells (hPSCs) or tissue-resident adult stem cells (ASCs) (Pek and Gu, 2024). When cultured under tightly controlled biomimetic conditions, these progenitor cells recapitulate the specific spatial architecture, cellular diversity, and functional complexity of the native pulmonary epithelium, effectively bridging the chasm between simplistic *in vitro* assays and complex human physiology (Chu et al., 2026). Recent bibliometric and scientometric analyses indicate a rapid increase in global lung organoid research. Since earlier studies on airway and alveolar epithelial stem/progenitor cell-derived three-dimensional culture systems, lung organoid research has expanded substantially over the past decade (Rock et al., 2009; Barkauskas et al., 2013; Wang et al., 2025b). This growth reflects the increasing recognition of organoid technology as a useful human-relevant platform for respiratory biology, disease modeling, and translational research.

The multifaceted utility of lung organoids extends far beyond basic developmental biology, actively permeating highly applied disciplines including precision disease modeling, personalized oncology, high-throughput pharmacological screening, and complex toxicological safety assessments (Han et al., 2024; Madan et al., 2025; Singh et al., 2025; Chu et al., 2026). The recent passage of the FDA Modernization Act 2.0 has catalyzed this transition, by reducing mandatory reliance on animal testing and allowing consideration of animal or non-animal methods, including cell-based assays, microphysiological systems, and organoids, depending on context-specific validation (Zushin et al., 2023; Williams, 2024). However, the current generation of benchtop lung organoids remains constrained by several profound translational hurdles. Chief among these are a lack of advanced structural maturation, the critical absence of perfusable vascular networks, limited integration with resident and circulating immune cells, and an overwhelming reliance on undefined, highly variable animal-derived extracellular matrices (Joo et al., 2024).

This review aims to provide an exhaustive, nuanced, and forward-looking synthesis of the latest advancements in lung organoid technology. We meticulously examine the developmental trajectories of these models and their deployment in decoding the pathogenesis of respiratory diseases. Furthermore, we systematically evaluate the existing translational barriers impeding clinical integration and highlight state-of-the-art bioengineering solutions—ranging from mechanobiologically tunable synthetic hydrogels to microfluidic organ-on-a-chip (OoC) platforms and precision 3D bioprinting techniques—that are absolutely essential for propelling these microphysiological systems toward broad, standardized clinical and industrial adoption.

## 2 Cellular origins, development, and derivation of lung organoids

The physiological fidelity, predictive power, and ultimate translational utility of lung organoids are intrinsically dictated by the developmental trajectory and cellular provenance of the founding stem cell populations (Moreira et al., 2022; Bell and Krasnodembskaya, 2025; Wang et al., 2025b). To accurately mimic the regional specificity of the human respiratory tract, current lung organoid models are primarily established from two distinct ontological sources: tissue-resident adult stem cells (ASCs) isolated from mature pulmonary tissue, and human pluripotent stem cells (hPSCs), which encompass both embryonic stem cells (ESCs) and artificially reprogrammed induced pluripotent stem cells (iPSCs) (van der Vaart and Clevers, 2021; Moreira et al., 2022; Chen et al., 2025). The strategic selection of the cellular source dictates the specific compartmental architecture modeled, yielding a diverse array of structural prototypes, including tracheospheres, bronchiospheres, alveolospheres, and multilineage bronchioalveolar constructs (Vazquez-Armendariz and Tata, 2023; Joo et al., 2024).

### 2.1 Pluripotent stem cell-derived organoid ontogeny

The generation of lung organoids from hPSCs represents a triumph of developmental biology, as it strictly mirrors the sequential, spatially restricted, and temporally defined milestones of embryonic lung organogenesis (Thangam et al., 2024). Under precisely orchestrated *in vitro* culture conditions, hPSCs are sequentially directed through a series of morphogen-driven fate decisions (Esfahani et al., 2021). The process initiates with the induction of definitive endoderm via potent Nodal/Activin A signaling, followed by the suppression of Wnt and BMP pathways to specify anterior foregut endoderm (AFE) (Thangam et al., 2024). Subsequent manipulation yields ventral anterior foregut endoderm cells (VAFECs) (Vazquez-Armendariz and Tata, 2023).

The critical inflection point in this directed differentiation cascade is the successful induction of specialized lung progenitor cells, commonly identified by NKX2.1 expression in combination with developmental context and additional markers such as FOXA2, SOX2, and SOX9, because NKX2.1 is also expressed in thyroid and forebrain lineages (Vazquez-Armendariz and Tata, 2023). These isolated NKX2.1+ progenitors represent the multipotent foundation capable of giving rise to the entire diverse repertoire of pulmonary epithelial lineages. Subsequent modulation using carefully titrated cocktails of Wnt, Fibroblast Growth Factor (FGF), Bone Morphogenetic Protein (BMP), and Retinoic Acid (RA) signaling pathways drives extensive branching morphogenesis and distal alveolar maturation during lung organoid differentiation (Vazquez-Armendariz and Tata, 2023; Joo et al., 2024; Thangam et al., 2024). This intricate process eventually yields complex, self-organizing bronchioalveolar organoids comprising a highly heterogenous mixture of basal, club, ciliated, goblet, and alveolar type 1 and 2 (AT1/AT2) cells (Thangam et al., 2024). The hPSC-derived platforms are particularly advantageous for interrogating inherited monogenic disorders via CRISPR/Cas9 precision genome editing, longitudinally tracking early developmental abnormalities, and generating patient-specific models for personalized medicine from accessible somatic cells such as dermal fibroblasts or peripheral blood mononuclear cells (Naffaa, 2025).

### 2.2 Adult stem cell-derived organoids and patient-specific modeling

In sharp contrast to hPSCs, ASCs are isolated directly from clinical patient biopsies, surgical resections, or bronchoalveolar lavage fluid in respiratory organoid studies (Sachs et al., 2019; van der Vaart and Clevers, 2021; Joo et al., 2024). The paramount advantage of ASC-derived organoids—frequently termed patient-derived organoids (PDOs)—is their unique capacity to retain the distinct epigenetic signatures, chronological aging markers, and accumulated somatic mutational profiles of the original donor tissue (Chen and Na, 2022). This inherent biological memory makes ASCs the undisputed gold standard for modeling acquired, age-related, and environment-driven pathologies such as lung cancer and chronic inflammatory diseases (Tang and Tian, 2025).

Depending on the precise anatomical site of isolation, ASCs self-organize into highly specific regional models. Tracheospheres and bronchiospheres are primarily derived from resident basal stem cells (e.g., KRT5+, TP63+, NGFR + populations) and secretory club cells (SCGB1A1+), which can generate differentiated airway epithelial cell types such as ciliated cells (FOXJ1+) and goblet cells (MUC5AC+) that directly contribute to mucociliary clearance (Chu et al., 2026). Conversely, distal alveolar organoids are propagated from isolated AT2 cells, which function as the primary facultative stem cell pool of the delicate alveolar space (Kühl et al., 2023). When co-cultured with supportive lung mesenchymal fibroblasts to meticulously simulate the native multipotent stem cell niche, these AT2 cells robustly proliferate and sequentially differentiate into flattened, gas-exchanging AT1 cells, mirroring the delicate architectural interface of the distal lung (Ligresti et al., 2023). A comparative synthesis of these organoid modalities is detailed in Table 1.

**TABLE 1:** Classification, cellular origins, and representative disease modeling applications of distinct lung organoid platforms.

Organoid classification	Primary cellular origin and key molecular markers	Modeled respiratory pathologies and applications	References
Tracheospheres	Tracheal basal stem cells (KRT5+, TP63+, NGFR+)	Proximal airway infections, cystic fibrosis	Vazquez-Armendariz and Tata (2023)
Bronchiospheres/airway organoids	Bronchial progenitors: basal cells, club cells (SCGB1A1+)	COPD, Asthma, Viral Infections (Influenza, RSV, SARS-CoV-2)	Joo et al. (2024)
Alveolar organoids	Distal alveolar progenitors: AT2 cells (SFTPC+) co-cultured with mesenchyme	IPF, COVID-19 ARDS, Chemical Toxicity	Thangam et al. (2024)
Bronchioalveolar organoids	Dual airway-alveolar progenitor populations; hPSC-derived	Complex host-pathogen interactions, spatial developmental tracking	Joo et al. (2024)

## 3 High-fidelity disease modeling and translational drug discovery

**FIGURE 1:**
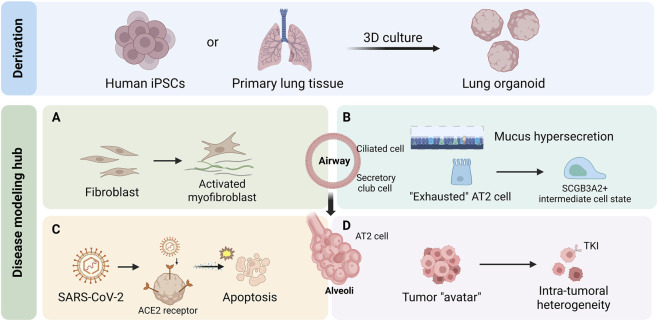
High-fidelity disease modeling and translational drug discovery. The figure summarizes the use of lung organoids in modeling major respiratory diseases, including COPD, IPF, respiratory viral infection, and lung cancer. It distinguishes proximal airway organoid models from distal alveolar organoid models and highlights key disease-relevant readouts, including epithelial differentiation, mucus production, ciliary function, viral entry-factor expression, fibrotic remodeling, and patient-specific drug response. **(A)** Idiopathic pulmonary fibrosis (IPF). **(B)** COPD & Asthma. **(C)** Infecctious disease. **(D)** Lung cancer.

The unprecedented structural fidelity, robust transcriptomic alignment, and functional complexity of lung organoids have catalyzed a monumental paradigm shift in the investigation of complex respiratory diseases (Joo et al., 2024). By meticulously preserving the intricate spatial relationships and dynamic cell-cell crosstalk inherent to human lungs, these 3D models provide an unparalleled window into fundamental pathogenesis, cellular senescence, and pharmacological responses, serving as a highly predictive bridge to clinical trials (Pentimalli et al., 2025) (Figure 1).

### 3.1 Idiopathic pulmonary fibrosis (IPF) and matrix remodeling

IPF is an aggressive, chronic, and life-limiting interstitial lung disease characterized by unrelenting myofibroblast activation, pathological stiffening of the extracellular matrix, and irreversible destruction of the alveolar architecture (Samarelli et al., 2021; Mei et al., 2022). The persistent failure of current anti-fibrotic therapies to halt or reverse disease progression highlights the profound inadequacy of traditional rodent bleomycin-induced fibrosis models, which spontaneously resolve and fail to mimic the progressive nature of human IPF (Yanagihara et al., 2020; Vazquez-Armendariz et al., 2022).

Lung organoids, particularly those incorporating both functional epithelial and resident mesenchymal compartments, can model selected human fibrogenic responses rather than fully recapitulating the complete progressive course of IPF (Vazquez-Armendariz et al., 2022; Zhu et al., 2025). Utilizing hPSC-derived alveolar organoids, investigators have longitudinally documented bleomycin-induced epithelial cellular senescence, robust fibroblast-to-myofibroblast transitions, and the dense, disorganized accumulation of collagenous extracellular matrix, ultimately leading to the visible macroscopic contraction of the entire organoid architecture (Suezawa et al., 2021). High-resolution single-cell RNA sequencing (scRNA-seq) of these fibrotic organoids reveals discrete, pathologically distinct cellular clusters that dynamically mimic *in vivo* fibrogenesis at the molecular level (Wang et al., 2025a). Crucially, these advanced 3D fibrotic models (frequently termed lung organoid-based fibrosis, LOFs) serve as highly sensitive, scalable platforms for systematically evaluating the efficacy, target engagement, and cytotoxicity of next-generation anti-fibrotic candidates prior to costly clinical entry (Wang et al., 2025a).

### 3.2 Chronic obstructive pulmonary disease (COPD) and asthma

COPD is characterized by progressive airflow limitation, chronic destructive bronchitis, and emphysematous alveolar degradation driven by protracted exposure to noxious airborne particles, primarily cigarette smoke (Hogg and Timens, 2009). Traditional models struggle immensely to replicate the chronic timeline and specific epithelial metaplastic remodeling observed in human COPD (Stolz et al., 2022). Lung organoids, however, have successfully simulated the complex pathophysiology of this devastating disease. Recent seminal studies utilizing alveolar organoids derived directly from COPD patients have observed spontaneous pathological transformations, such as the abnormal re-differentiation and exhaustion of functional AT2 cells into intermediate SCGB3A2+ states, directly mirroring the metaplastic shifts observed in clinical patient biopsies and previously reported transitional epithelial states (Kathiriya et al., 2022; Huo et al., 2025).

Furthermore, researchers have leveraged organoids to identify and interrogate critical genetic vulnerabilities, such as the Family with Sequence Similarity 13 Member A (FAM13A) gene (Huo et al., 2025). Genome-wide association studies (GWAS) have heavily implicated FAM13A in COPD susceptibility. Organoids genetically engineered to lack FAM13A exhibit significantly hyper-proliferative and aberrant differentiation phenotypes upon chronic exposure to cigarette smoke extract, powerfully illuminating the molecular basis of epithelial dysfunction and providing a novel target for pharmacological intervention (Gong et al., 2021). For asthma and other hypersecretory obstructive conditions, airway organoids enable the precise quantification of mucus hypersecretion, ciliary dyskinesia, and barrier disruption, facilitating the rapid, high-throughput screening of novel bronchodilators, mucolytics, and epithelial-targeted therapeutic candidates (Zhou et al., 2023).

### 3.3 Infectious diseases, host-pathogen interactions, and pandemic preparedness

The inherent vulnerability of the human respiratory tract to aerosolized airborne pathogens makes lung organoids indispensable, front-line tools for infectious disease biology. Prior to the 2020 pandemic, immortalized, highly mutated cell lines such as A549, Calu-3, and VeroE6 were the global standard for viral propagation; however, they exhibit heavily skewed, non-physiological viral entry mechanisms and lack intact endogenous immune signaling pathways (Yao et al., 2020). The COVID-19 pandemic forcefully underscored the urgent necessity for physiologically relevant, human-specific models for respiratory virus research (Edwards et al., 2022; Li et al., 2025b).

Bronchial and alveolar organoids can express critical host entry factors—such as the ACE2 receptor and TMPRSS2 protease—although their expression levels and localization may vary depending on organoid source, differentiation state, and culture conditions (Edwards et al., 2022). Studies employing bi-potential respiratory organoids demonstrated productive SARS-CoV-2 infection with variant- and cell-type-dependent differences in viral replication and host response, particularly for the highly evasive Omicron variant (Li et al., 2025b). Infection elicited rapid, epithelial-intrinsic inflammatory cytokine responses and severe cellular apoptosis that strikingly mirrors the clinical progression of acute respiratory distress syndrome (ARDS) observed in severe COVID-19 patients (Li et al., 2025b). Beyond coronaviruses, organoids are actively and extensively utilized to chart the infectivity, tissue tropism, and cytopathic effects of influenza viruses (revealing details of virion endocytosis), respiratory syncytial virus (RSV) bronchiolitis, parainfluenza, and intracellular bacterial pathogens such as *Mycobacterium tuberculosis*. Expanding global access to these advanced infection-competent organoid platforms, preventing technological monopolization by high-income nations, is increasingly recognized by health policymakers as a critical pillar of proactive future pandemic preparedness (Hossain and Kim, 2025).

### 3.4 Lung cancer, tumor microenvironments, and precision oncology

Non-small cell lung cancer (NSCLC)—comprising adenocarcinoma, squamous cell carcinoma, and large cell neuroendocrine carcinoma—exhibits profound intra-tumoral heterogeneity and massive genomic instability, driving rapid therapeutic resistance and staggering clinical mortality rates (Kim et al., 2026). Patient-derived organoids (PDOs) have revolutionized the paradigm of personalized oncology by acting as highly faithful, *ex vivo* “avatars” of the patient’s primary tumor (Lee et al., 2025). Unlike traditional patient-derived xenografts (PDXs), which are notoriously slow to establish, cost-prohibitive, and suffer from eventual murine stromal replacement, NSCLC PDOs may be established within a clinically relevant timeframe, but establishment success varies by tumor subtype, sample quality, culture condition, and the risk of normal airway organoid overgrowth (Dijkstra et al., 2020; Tang and Tian, 2025). Crucially, they preserve the exact genetic mutational landscape, phenotypic architecture, structural heterogeneity, and pharmacological drug sensitivity profiles of the individual patient (Yin et al., 2025), although they may lack stromal, vascular, and immune components unless additional co-culture or microphysiological strategies are incorporated.

Extensive PDO libraries have been instrumental in delineating elusive mechanisms of clinical resistance to targeted therapies, such as epidermal growth factor receptor tyrosine kinase inhibitors (EGFR-TKIs). For instance, high-throughput organoid screening recently identified DCLK1-dependent Wnt/β-catenin signaling activation as a hidden bypass track for TKI resistance, thereby unveiling novel, highly synergistic combinational therapies for refractory patient populations (Yan et al., 2022). Furthermore, investigations utilizing lung adenocarcinoma-derived organoids have mapped out the functional consequences of specific mutations in genes such as GSTP1 and BRMS1, paving the way for hyper-targeted precision therapeutics (Meng et al., 2025).

## 4 Next-generation predictive toxicology and safety assessment

**FIGURE 2:**
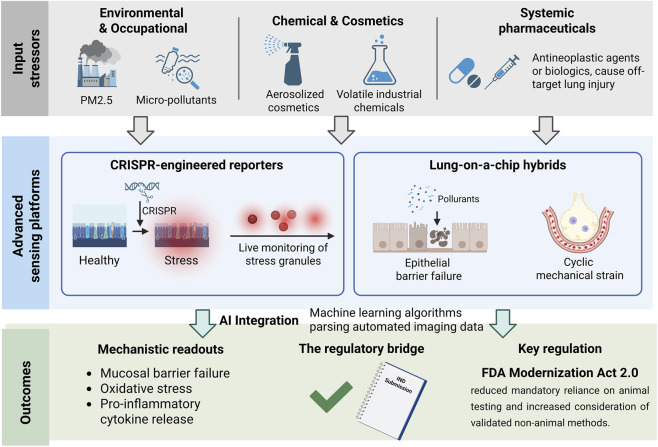
Next-Generation Predictive Toxicology and Safety Assessment. The figure summarizes the use of lung organoids and lung-on-a-chip systems for evaluating inhaled pollutants, chemical stressors, cosmetic aerosols, and drug-induced pulmonary toxicity. Major readouts include epithelial barrier disruption, oxidative stress, inflammatory signaling, cell death, and altered differentiation. The figure also indicates the regulatory interest in validated New Approach Methodologies while avoiding the implication that organoids are automatically accepted as replacements for animal models.

Beyond accelerating therapeutic discovery, lung organoids are profoundly disrupting and modernizing the fields of predictive toxicology and safety pharmacology (Figure 2). The delicate pulmonary epithelium is the initial site of toxicological insult following inhalation, necessitating robust models that can accurately predict human inflammatory and apoptotic responses to exogenous stimuli (Kastlmeier et al., 2022).

### 4.1 Environmental and occupational inhalation toxicity

Airborne particulate matter (PM), notably fine PM2.5, constitutes a ubiquitous global environmental hazard causally linked to transient lung injury, severe COPD exacerbation, cardiovascular disease, and pulmonary carcinogenesis (Kang et al., 2025). Evaluating the intricate toxicodynamics of complex urban aerosols, industrial diesel exhaust, and emerging micro-pollutants like nanoplastics and tire wear particles (TWPs) is exceptionally challenging in static 2D systems, which cannot model mucosal clearance or multi-cellular barrier integrity (Kang et al., 2025).

Recent studies employing 3D human airway organoids to assess the toxicity of TWPs revealed significant disruption of respiratory mucosa integrity (Jiang et al., 2023). Exposure induced profound intracellular oxidative stress, triggered the release of inflammatory cytokines, and caused the marked distortion and depletion of vital epithelial progenitor stem cell pools in organoid-based inhalation toxicology models (Jiang et al., 2023; Kang et al., 2025). These sophisticated 3D models demonstrate markedly higher sensitivity to toxicant exposure than traditional monolayer cultures or even certain *in vivo* assays, providing granular mechanistic insights into exactly how fine particulate matter breaches the alveolar barrier (Dave et al., 2025).

### 4.2 Chemical hazard and cosmetic safety assessment

The stringent safety evaluation of aerosolized cosmetic products, domestic chemical disinfectants, and volatile industrial chemicals has historically relied heavily on extensive, costly, and ethically contentious *in vivo* animal testing. However, increasing global ethical scrutiny and rigid international legislative bans on animal testing for cosmetics demand the rapid deployment of robust, human-relevant alternatives. Lung organoids offer a biologically sophisticated, highly reproducible testing matrix for calculating safe exposure thresholds and performing initial hazard identification (Li et al., 2025c).

To meticulously track dynamic molecular responses to chemical stressors in real time, researchers have innovatively engineered hPSC-derived lung organoids using CRISPR/Cas9 technology to express endogenous fluorescent reporters, such as G3BP1-mCherry (Kim et al., 2026). This genetic modification allows for the non-destructive, continuous live visualization of stress granule (SG) formation—an early, sensitive indicator of cellular distress that significantly precedes overt epithelial barrier failure and cellular apoptosis (Kim et al., 2026). This reporter-based strategy provides one example of how lung organoids can be incorporated into Next-Generation Risk Assessment (NGRA), but broader toxicological application still requires additional assays, standardized exposure protocols, and independent validation.

### 4.3 Drug-induced lung injury (DILI) and evolving regulatory paradigms

Systemic therapeutics, including novel antineoplastic agents, biologics, conventional disease-modifying antirheumatic drugs (DMARDs), and antiarrhythmics, frequently exhibit unpredictable, idiosyncratic pulmonary toxicity (Bridi et al., 2024). This culminates in life-threatening Drug-Induced Lung Injury (DILI), a highly heterogeneous clinical condition characterized by organizing pneumonia, diffuse alveolar damage, and acute respiratory failure (Bridi et al., 2024). The pathogenesis of DILI is variable and multifactorial, making it notoriously difficult to predict using standard animal models (Fitzpatrick et al., 2024).

The critical transition away from reliance on animal models is legally formalized by the recent FDA Modernization Act 2.0, which reduces mandatory reliance on animal testing and allows animal or non-animal methods to be considered in drug development, depending on regulatory context and validation status (Zushin et al., 2023). While microfluidic liver chips have successfully set powerful regulatory precedents by accurately predicting drug-induced hepatotoxicity with over 87% accuracy in a retrospective study reported by Ewart *et al.* (Ewart et al., 2022), comparable validation remains necessary for lung organoids and lung-on-a-chip systems before they can be considered reliable predictors of respiratory DILI. When appropriately validated, these platforms may help identify selected human-specific pulmonary toxicities earlier in drug development. Table 2 outlines key contemporary applications of lung organoid systems in toxicology.

**TABLE 2:** Critical applications of advanced lung organoid models in predictive toxicology and inhalation safety assessment.

Toxicant/Exogenous stressor	Specific organoid model utilized	Primary toxicological findings and endpoints	References
Urban particulate matter (PM2.5)	hESC-derived alveolar organoids; patient airway organoids	Mucosal barrier disruption, robust induction of oxidative stress, transient tissue injury	Kang et al. (2025)
Emerging pollutants (tire wear particles)	Human airway organoids (hAOs)	Severe barrier dysfunction, aberrant dysregulation of epithelial stem cell progenitors	Jiang et al. (2023)
Aerosolized cosmetics	3D stem-cell derived respiratory organoids	Initial hazard identification; potential alternative to selected *in vivo* inhalation testing	Li et al. (2025c)
Chemical stressors & disinfectants	CRISPR-engineered (G3BP1-mCherry) reporter organoids	Rapid, quantifiable stress granule (SG) dynamics fundamentally preceding epithelial injury	Kim et al. (2026)
Systemic therapeutics (e.g., antineoplastics)	Multi-cellular organoids and integrated Lung-on-a-Chip	Clinical concern: drug-induced lung injury, including organizing pneumonia and diffuse alveolar damage; model status: emerging platforms requiring further validation for predictive DILI assessment	Bridi et al. (2024), Fitzpatrick et al. (2024)

## 5 Overcoming translational hurdles via advanced bioengineering

Despite demonstrating undeniable, transformative superiority over simplistic 2D cultures, standard static benchtop lung organoids possess significant physiological limitations that critically obstruct their seamless integration into routine clinical translation and industrial-scale pharmacology (Joo et al., 2024). To surmount these formidable barriers, the field is undergoing a rapid, necessary transition from basic cellular biology to multidisciplinary tissue engineering.

### 5.1 The missing microenvironment: the vascularization and immune integration bottleneck

The most prominent functional deficit in conventional organoid models is the absence of a perfusable, functional vascular network (Zhang et al., 2021). Native human lung tissue is exquisitely vascularized, forming an intimate capillary-alveolar interface that is absolutely essential for efficient gas exchange, continuous nutrient delivery, and metabolic waste removal. *In vitro*, as static organoids mature and expand beyond diffusion limits typically around 100–200 μm from a nutrient source, simple passive diffusion becomes grossly inadequate to support the dense, highly active cellular core (Wang et al., 2026). This severe nutrient deprivation results in a pronounced, expanding necrotic core, severely truncating the long-term viability of the culture and entirely precluding the longitudinal study of chronic disease progression (Nwokoye and Abilez, 2024). Furthermore, the lack of interconnected vasculature prevents the systemic modeling of circulating drug pharmacokinetics, metastatic cellular dissemination, and leukocyte extravasation (Roy and Cucullo, 2026).

Equally critical to the translational bottleneck is the lack of a competent, interacting endogenous immune system. Human lungs harbor a vast, complex array of resident alveolar macrophages, dendritic cells, and innate lymphoid cells that orchestrate responses to invading pathogens, emerging tumors, and fibrotic stimuli (Du et al., 2023). Current rudimentary methods attempt to force immune integration by manually co-culturing exogenous peripheral blood mononuclear cells (PBMCs) or autologous patient-derived immune cells with the epithelial organoids (Yip et al., 2023). However, these artificial co-cultures face severe, often insurmountable limitations: culture medium incompatibility often induces unintended baseline immune activation or rapid cellular apoptosis, the functional half-life of endogenous immune components *in vitro* is extremely short, and these models frequently fail to recapitulate the precise, physiological spatial distribution of immune cells within the complex tumor or fibrotic microenvironment (Huang et al., 2021).

### 5.2 Matrix undefinedness: moving beyond the matrigel conundrum

The structural scaffold universally employed for initial organoid derivation and growth is Matrigel (or equivalent basement membrane extracts, BME)—an undefined, gelatinous protein mixture secreted by Engelbreth-Holm-Swarm mouse sarcoma cells (Joo et al., 2024). While Matrigel provides a potent, laminin-rich environment highly conducive to initial stem cell expansion, its xenogeneic (animal) origin introduces profound, unacceptable batch-to-batch variability (Marfoglia and Sorrentino, 2025). The presence of unquantified, highly variable murine growth factors and morphogens severely confounds precise mechanistic signaling studies and introduces immense statistical noise into high-throughput drug screens (Zhang et al., 2025b).

More critically, the animal origin of Matrigel poses a hard, insurmountable regulatory barrier against utilizing these organoids in direct regenerative medicine applications due to the severe risk of xenogeneic pathogen transfer and profound immunogenicity upon human transplantation (Huang et al., 2021). To phase out this reliance on undefined murine matrices, biomaterials researchers are developing fully defined, highly tunable synthetic and natural polymeric hydrogels (Zhang et al., 2025b). Synthetic polymers, such as polyethylene glycol (PEG), alongside natural biopolymers like alginate, nanocellulose, and gelatin methacryloyl (GelMA), permit the precise, independent decoupling of biochemical ligands from bulk mechanical properties (Roy and Cucullo, 2026). Poly (lactide-co-glycolide) (PLGA)-based porous degradable scaffolds may also be used in tissue engineering, but they are distinct from hydrogel matrices. In native fibrotic diseases such as IPF, the mechanical stiffness of the lung tissue increases dramatically. Tunable synthetic hydrogels allow researchers to physically manipulate matrix stiffness (e.g., dynamically increasing stiffness to >1.5 kPa) to actively induce downstream mechanotransduction pathways, thereby triggering robust fibroblast activation purely via physical cues, without the need for exogenous chemical profibrotics (Vazquez-Armendariz et al., 2022). Furthermore, the utilization of decellularized extracellular matrix (dECM) hydrogels derived directly from human lung tissue offers a highly biomimetic, tissue-specific proteomic signature without the xenogeneic variability of Matrigel (Zhang et al., 2025b).

### 5.3 Establishment and standardization of lung organoid cultures

Despite their utility, lung organoid systems still face challenges in establishment efficiency, purity, long-term maintenance, and standardization. Long-term expanding human airway organoids have provided useful models for disease modeling, but culture conditions, tissue source, passage number, and matrix composition can influence reproducibility (Sachs et al., 2019). For lung cancer organoids, normal airway organoid overgrowth may limit the establishment of pure tumor organoids, emphasizing the need to verify tumor purity and genomic concordance before personalized drug testing (Dijkstra et al., 2020). Recent two-step protocols for lung cancer primary 3D cultures further highlight the importance of distinguishing short-term primary cultures from long-term expandable organoid systems (Strocchi et al., 2024). Therefore, standardized reporting of sample source, culture medium, extracellular matrix, passage number, establishment success rate, and quality-control assays remains essential for translational application.

### 5.4 Microphysiological systems: organ-on-a-chip (OoC) integration

Organoid-on-a-chip (OoC) platforms elegantly and comprehensively resolve the static limitations of traditional petri dish cultures by introducing continuous, dynamically controlled microfluidic perfusion (Li et al., 2024). Lung-on-a-chip devices are typically fabricated using flexible, biocompatible polydimethylsiloxane (PDMS) and consist of separated, parallel microchannels designed to explicitly model the delicate alveolar-capillary interface. However, PDMS can absorb hydrophobic small-molecule drugs, which may alter effective compound concentrations and affect pharmacokinetic or toxicological readouts. By precisely seeding lung organoid-derived epithelial cells on an upper porous membrane and human vascular endothelial cells on the lower face, researchers establish a functional air-liquid interface (ALI) that is absolutely critical for proper, mature lung differentiation and mucociliary function (Joo et al., 2024).

Crucially, OoC devices allow for the application of cyclic mechanical strain—achieved by applying rhythmic, controlled vacuum pressure to lateral flexible chambers—which exquisitely and faithfully mimics the physical 3D stretching of alveoli during normal human respiration (Joo et al., 2024). This mechanical biophysical stimulus is indispensable for accelerating cellular maturation, enhancing natural surfactant production, and regulating tight-junction barrier permeability. The integration of built-in, non-invasive biosensors permits the real-time, temporal monitoring of transepithelial electrical resistance (TEER), effectively tracking barrier integrity and evaluating the precise kinetics of nanoparticle, drug, or PM2.5 translocation from the simulated airway compartment directly into the simulated bloodstream (Roy and Cucullo, 2026). Advanced iterations now involve interconnected multi-organ chips such as gut-liver, liver-kidney, and heart-liver systems to study systemic drug metabolism and multi-organ toxicity simultaneously (Roy and Cucullo, 2026). Organ-on-a-chip systems have also been applied to radiation-induced lung injury, which remains difficult to study using conventional experimental models. A human lung alveolus-on-a-chip model of acute radiation-induced lung injury has been developed to examine epithelial-endothelial injury responses under controlled microphysiological conditions (Dasgupta et al., 2023).

### 5.5 3D bioprinting, sacrificial inks, and multilineage assembloids

Three-dimensional bioprinting offers unparalleled spatial resolution, enabling the deterministic, highly controlled placement of diverse cells, immune components, and specialized matrix exactly where required, addressing the randomness of spontaneous self-organization (Yoon et al., 2025). To directly resolve the vascularization bottleneck, bioengineers employ sophisticated sacrificial bioinks, such as Pluronic F127 or thermally sensitive gelatin, printed in complex, fractal-like branching patterns within a primary lung hydrogel scaffold (Nwokoye and Abilez, 2024). Following the stable gelation of the bulk matrix, the sacrificial ink is thermally liquefied and evacuated, leaving behind hollow, interconnected microchannels that are subsequently seeded with human endothelial cells (e.g., HUVECs or iPSC-ECs) to form mature, fully perfusable vascular networks (Unagolla and Jayasuriya, 2022).

Such perfusable architectures not only prevent core necrosis in massive, centimeter-scale organoid constructs but also permit the dynamic systemic infusion of circulating immune cells, accurately replicating physiological monocyte extravasation and complex inflammatory cascades *in vitro* (Yang et al., 2020). Furthermore, high-resolution drop-on-demand inkjet bioprinting has recently achieved the single-step fabrication of thin alveolar barrier-like structures complete with microfluidic capabilities, representing a technological improvement over manual, artificially thick biofabrication techniques (Li et al., 2022).

Recent developmental innovations also include the generation of multilineage “assembloids.” By meticulously controlling mesoderm-endoderm co-differentiation via highly precise BMP signaling modulation, researchers have successfully generated entirely self-vascularizing multilineage organoids. Upon *in vivo* xenotransplantation under the kidney capsule of murine models, these pre-vascularized organoids rapidly and seamlessly anastomose with the host circulatory system, unlocking extensive growth, architectural complexity, and full functional maturation that was previously deemed impossible in static *in vitro* culture (Matsumoto et al., 2021).

## 6 Conclusion and future perspectives

The rapid, exponential evolution of lung organoid technology from rudimentary, static cellular spheres into highly sophisticated, dynamically bioengineered microphysiological systems marks a pivotal and irreversible era in respiratory research (Chu et al., 2026). By faithfully mimicking the dynamic cellular complexity, mutational landscapes, and architectural nuance of human lungs, organoids are systematically dismantling the translational hurdles that have long plagued pulmonary pharmacology and toxicology (Zhou et al., 2023). As global regulatory bodies—spurred by initiatives like the FDA Modernization Act 2.0—increasingly embrace New Approach Methodologies (NAMs) to phase out ethically and scientifically flawed animal testing, the demand for standardized, scalable, and highly predictive organoid assays will only accelerate across both academia and the pharmaceutical industry.

Looking forward, the seamless convergence of multi-omics profiling (including single-cell RNA sequencing, spatial transcriptomics, and advanced proteomics) with organoid biology will dramatically deepen our understanding of rare cellular subpopulations and dynamic, multi-pathway drug-disease interactions at unprecedented, single-cell resolutions (Wang et al., 2025a). Furthermore, the integration of artificial intelligence (AI), machine learning algorithms, and automated high-throughput robotic liquid handling systems will actively mitigate the persistent reproducibility issues associated with manual culture (Rashid and Kausik, 2024). By standardizing organoid generation and rapidly parsing massive, complex streams of multi-dimensional imaging and sensor data from organ-on-a-chip platforms, these AI-driven workflows may contribute to future patient-specific predictive frameworks, including digital twin-like models, although such applications in respiratory organoid research remain exploratory and require rigorous validation (Roy and Cucullo, 2026).

However, the path to ubiquitous clinical translation requires actively addressing ongoing ethical, logistical, and socioeconomic challenges. The establishment of massive, highly annotated patient-derived biobanks mandates rigorous ethical oversight regarding genetic data privacy, informed patient consent, and data ownership (Yin et al., 2025). Moreover, democratizing access to these advanced bioengineering platforms is critical. Currently, the expertise and capital required to develop advanced OoC and bioprinting systems are concentrated heavily in the United States and Europe, although China and other rapidly growing research communities have also made substantial contributions to organoid research (Yin et al., 2025). Preventing the monopolization of this technology and ensuring equitable global access is an urgent international health imperative, particularly for fortifying worldwide preparedness against future, inevitably emerging respiratory pandemics (Yin et al., 2025).

In conclusion, lung organoids have substantially advanced the landscape of respiratory disease modeling, offering a high-fidelity, human-centric alternative to conventional animal models and simplistic two-dimensional cultures. From identifying novel genetic drivers in COPD and evaluating anti-fibrotic efficacy in IPF, to charting the rapid infectivity of novel respiratory viruses and predicting insidious drug-induced lung injuries, these 3D models provide a useful platform for both fundamental basic discovery and advanced, regulatory-grade safety assessments. While significant biological limitations persist—chiefly regarding vascularization, immune integration, and structural maturation—the strategic application of cutting-edge bioengineering principles provides clear, highly actionable pathways forward. Through the synergistic implementation of programmable synthetic hydrogels, dynamic microfluidic perfusion systems, and high-precision 3D bioprinting, the scientific community is successfully engineering the vital microenvironmental cues necessary to realize the full translational potential of lung organoids. Continued interdisciplinary innovation, together with standardized protocols and context-specific validation, will be necessary to support the broader use of these platforms in personalized respiratory medicine and next-generation predictive toxicology.
